# A Functional Misexpression Screen Uncovers a Role for *Enabled* in Progressive Neurodegeneration

**DOI:** 10.1371/journal.pone.0003332

**Published:** 2008-10-08

**Authors:** Carolina Rezával, Jimena Berni, Ezequiel Axel Gorostiza, Santiago Werbajh, María Marta Fagilde, María Paz Fernández, Esteban J. Beckwith, Ezequiel J. Aranovich, Carmen A. Sabio y García, María Fernanda Ceriani

**Affiliations:** Laboratorio de Genética del Comportamiento, Fundación Instituto Leloir, Instituto de Investigaciones Bioquímicas-Buenos Aires (IIB-BA, CONICET), Buenos Aires, Argentina; Massachusetts General Hospital/Harvard Medical School, United States of America

## Abstract

*Drosophila* is a well-established model to study the molecular basis of neurodegenerative diseases. We carried out a misexpression screen to identify genes involved in neurodegeneration examining locomotor behavior in young and aged flies. We hypothesized that a progressive loss of rhythmic activity could reveal novel genes involved in neurodegenerative mechanisms. One of the interesting candidates showing progressive arrhythmicity has reduced *enabled* (*ena*) levels. *ena* down-regulation gave rise to progressive vacuolization in specific regions of the adult brain. Abnormal staining of pre-synaptic markers such as *cystein string protein* (CSP) suggest that axonal transport could underlie the neurodegeneration observed in the mutant. Reduced *ena* levels correlated with increased apoptosis, which could be rescued in the presence of p35, a general Caspase inhibitor. Thus, this mutant recapitulates two important features of human neurodegenerative diseases, i.e., vulnerability of certain neuronal populations and progressive degeneration, offering a unique scenario in which to unravel the specific mechanisms in an easily tractable organism.

## Introduction

Age is a major risk factor for neurodegenerative diseases such as Alzheimer's (AD), Parkinson's (PD) and Huntington's (HD), which cause a terrible human toll. These pathologies share specific features such as late onset and progressive degeneration of specific neuronal populations. As society ages, neurodegenerative diseases will become increasingly common. Recent estimates claim that about 25 million people worldwide suffer from these devastating diseases, and these figures will double every 20 years to reach 81 millions by 2040 [1]. These startling statistics clearly underscore a need to understand the basic molecular and cellular processes underlying these disabling disorders.

*Drosophila* has provided a powerful genetic system in which to elucidate fundamental cellular pathways in the context of a developing and functioning nervous system. A number of fly models expressing genes associated to neurodegenerative diseases, such as *huntingtin*, α-*synuclein*, *ataxin* and *tau* have been generated [2], and genetic enhancers/suppressors were identified [3]–[7].

The initial forward genetic screens searched for mutants with reduced lifespan, which were then examined for morphological signs of degeneration [8]–[11]. Such endeavors were clearly time-consuming and meant to identify genes causing gross changes in brain morphology. Subsequently, additional genetic screens were carried out looking for histological signs of degeneration on mutants originally isolated on the basis of conditional paralytic phenotypes [12]–[14]. An alternative to the analysis of mutations leading to loss-of-function or dominant negative phenotypes, which are the most likely outcomes of chemical mutagenesis, is the generation of gain of function mutations [15]. This can be accomplished using a binary system such as GAL4/UAS (UAS, Upstream Activating Sequence) [16]. Misexpression screens thus provide a venue to identify genes that when overexpressed, misexpressed (i.e. ectopically), or are down-regulated in a restrictive pattern give rise to a novel phenotype [17], [18]. In addition, this strategy overcomes a clear limitation of loss-of-function screens in that it allows the identification of genes that generate lethality earlier in development, through restricting misexpression to a specific tissue or group of cells.

We have developed a novel genetic screen to identify genes associated to neurodegeneration based on the concept that behavior provides a reliable readout of the state of the underlying neuronal circuit. Our hypothesis is that altered gene expression leading to impaired neuronal homeostasis on the circuit underlying circadian behavior will in time result in progressive arrhythmicity. Thus, we targeted misexpression of endogenous genes within the circuit controlling locomotor activity through the GAL4/UAS system. Over 1000 novel EP [15] lines were generated in our laboratory. Young and aged individuals with restricted misexpression of an endogenous gene were directly compared looking for signs of degeneration. Thus, a P-element insertion associated with a progressive behavioral phenotype was identified and resulted in GAL4-mediated downregulation of the *enabled* locus. *ena* overexpression in the mutant background specifically rescued the behavioral phenotype. Reduced *ena* levels triggered degeneration in the brain; interestingly, only the optic lobe was particularly vulnerable to *ena* downregulation, developing aged-dependent vacuolization, which correlated with a progressively poorer performance in a phototaxis behavioral paradigm. We further showed abnormal staining of pre-synaptic markers suggesting that ENA deregulation may result in axonal clogging. Consistent with the observation of increased apoptosis in aged flies, expression of *p35* rescued the locomotor and structural defects of the mutant. Our results implicate the ENA/VASP family members in keeping postmitotic neuronal homeostasis.

## Results

### Age-associated changes in circadian behavior

**Figure 1:**
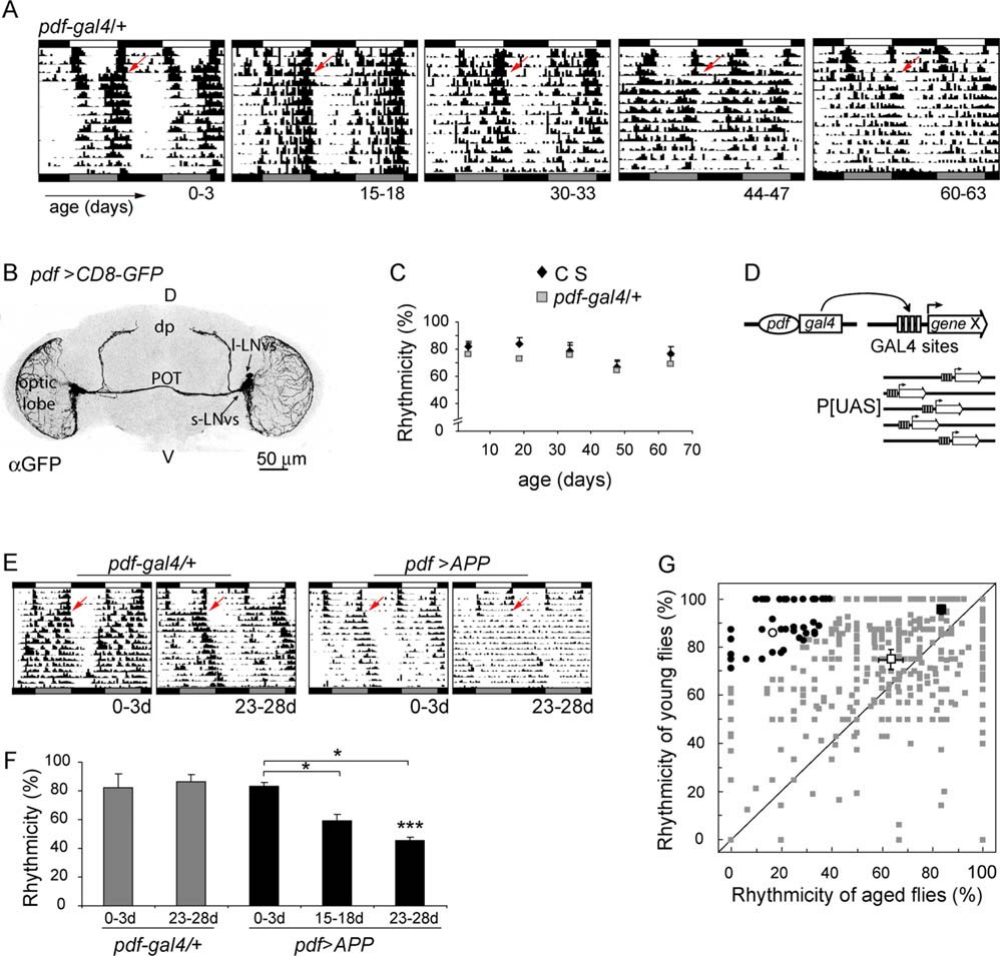
(A–C) Age-related changes in locomotor behavior. Pattern of rest/activity cycles at different times during adulthood evaluated in control lines. (A) Representative double plotted actograms from *pdf-gal4/+* flies of increasing age are shown. Each panel depicts the activity of a single fly throughout the experiment. Age at the onset of the experiment is indicated at the bottom of each panel. White, grey and black boxes indicate day, subjective day and night, respectively; arrows represent the transfer to constant darkness (DD). (B) Expression pattern of *pdf-gal4* driving a UAS-*CD8-GFP* reporter gene in the adult brain. GFP recreates the entire PDF circuit. Several relevant landmarks in the adult brain are indicated: small and large LNvs (s-LNvs and l-LNvs, respectively), posterior optic tract (POT), dorsal protocerebrum (dp). (C) Percentage of rhythmic flies for control genotypes (Canton S, CS, and *pdf-gal4/+*). No significant differences were found through the evaluated timescale. Data represent the mean±S.E.M. Statistical analysis was performed as described in experimental procedures. Three independent experiments were carried out. Additional details are included in Table S1. (D–G) A misexpression screen in young and aged flies reveals progressive behavioral phenotypes. (D) Schematic diagram of the misexpression screen. The *pdf-gal4* line was crossed to a number of independent target P[UAS] lines. In the progeny containing both elements the GAL4 transcription factor binds to the UAS within the P[UAS] transposon, inducing the misexpression of the gene immediately adjacent to it. (E–F) APP overexpression leads to progressive arrhythmicity. (E) Representative double plotted actograms of progressively older *pdf*>*APP* and control (*pdf-gal4*/+) flies. (F) The percentage of rhythmic flies for each strain is shown. Aged *pdf*>*APP* flies show decreased rhythmicity, which is significantly different from aged controls (***), and its younger counterparts (*) . (* *p*<0.05 and *** *p*<0.001). Three independent experiments including forty to seventy flies were carried out. (G) A direct comparison of rhythmicity for each misexpressed line in young and old flies. Misexpression of most P[UAS] lines do not result in a progressive phenotype (╦). Each dot represents the average rhythmicity for a certain insertion line tested at a young and older age. Flies that were highly rhythmic when young but whose rhythmicity decreased severely as they aged were considered as potential neurodegenerative mutants and further retested (indicated by •). This was the case with P[UAS]^117^ (○). Control flies such as *pdf-gal4/+* (□) and *pdf-gal4*, UAS-*CD8-GFP/++* (▪) describe the behavior of the majority of the lines tested (╦).

The extensive characterization of the neuronal circuit underlying circadian behavior makes it an ideal venue to search for mutations triggering neuronal dysfunction. This circuit includes eight neurons per brain hemisphere, four small and four large ventral Lateral Neurons (LNvs), which specifically express a neuropeptide called *pigment dispersing factor* (PDF, Fig. 1B) [19]. It has been shown that this circuit is central to the control of rhythmic activity [20].

Young flies are generally active around dawn and dusk, both under light-dark cycles and constant conditions. Since the focus of our work is to employ this behavior as a readout of neurodegeneration, we examined the pattern of rest/activity cycles at different stages during adult life in several control lines scoring a set of circadian parameters. Figure 1A includes a representative actogram of progressively older heterozygous *pdf-gal4* flies bearing a single copy of the driver employed in the genetic screen. Two commonly used wild type strains were examined in parallel (Canton S and *y w*, Table S1 and data not shown). Most parameters (periodicity, percentage of rhythmicity, overall activity) stayed relatively constant throughout the lifespan. In fact, the percentage of rhythmicity was only subtly affected as the flies aged (30 days and older; Fig. 1A, C and Table S1), although visual inspection suggests a clear loss of consolidation of the bouts of activity during the subjective day (compare left and right actograms in Fig. 1A); however this deconsolidation did not obscure the underlying rhythmicity assessed by periodogram analysis (Fig. 1C). Accordingly, the power of rhythmicity and the total locomotor activity tended to decrease in old files it has been described for other behavioral paradigms [21], without reaching statistical significance, while period length showed a tendency to increase reminiscent of what has been reported for other model systems [22].

In sum, rhythmicity was selected as the readout for neurodegeneration-associated changes since although its normal age related decline is subtle, impairment of this neuronal circuit has a robust impact on this behavior [23]. Thus, three-week old flies were selected to search for progressive phenotypic alterations since this time point gave robust activity and rhythmicity in controls (Fig. 1C; Table S1).

### A functional genetic screen retrieves genes potentially involved in neurodegeneration

To identify genes involved in neurodegeneration through gene deregulation without affecting the viability of the organism we altered the properties of the circadian system by means of the transgenic line *pdf-gal4* [24]. To first test the notion that neurodegeneration could lead to progressive arrhythmicity *amyloid precursor protein* (APP) expression was directed to the circadian circuit (*pdf*>*APP*). APP overexpression has been employed in fly models of AD [25], [26]; moreover, altered circadian patterns of activity have been reported in the APP23 mouse model, further strengthening this possibility [27]. Interestingly, a significant reduction in rhythmicity was observed as the flies progressively aged (Fig. 1E–F), thus validating this behavioral readout.

The *pdf-gal4* line was then employed to drive expression of independent transgenic insertions generated in our laboratory (Fig. 1D) from a founder P[UAS] line [15]. A comparison of the degree of rhythmicity of newly eclosed and 3-week-old flies was employed to identify genes potentially causing progressive neuronal dysfunction. The time frame was selected to ensure that most wild type flies would show no age-associated behavioral defects. Misexpressed lines showing arrhythmicity as young individuals were discarded from the analysis, since they are likely to pinpoint to deregulated circadian clock components. Roughly ten percent of the misexpressed insertions displayed progressive defects in rhythmic behavior, that is, highly rhythmic young individuals which become arrhythmic by three weeks of age (highlighted in black circles in Fig. 1G). Insertions displaying such robust age-dependent arrhythmycity were selected for reassessment of the phenotype and further characterization.

**Figure 2 pone-0003332-g002:**
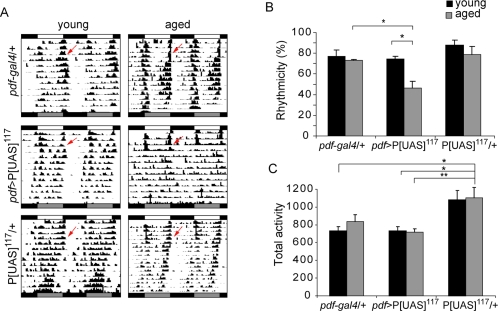
A novel P[UAS] insertion line shows progressive behavioral defects. Crossing P[UAS]^117^ to the *pdf-gal4* driver results in a significant decrease in the rhythmicity of old flies. (A) Representative double plotted actograms for young (3d) and aged (21d) flies of *pdf-gal4/* P[UAS]^117^ along with the corresponding controls. (B) The percentage of rhythmic flies for each strain is shown. Older *pdf-gal4/* P[UAS]^117^ flies are significantly different from their younger counterparts and from the aged controls (* *p*<0.05). Experiments were repeated at least three times. Additional details are included in Table S2. (C) No progressive decrease in daily activity was observed in either line. Although no differences were found between *pdf-gal4/* P[UAS]^117^ and *pdf-gal4/*+ individuals, young and aged heterozygous P[UAS]^117^ flies were overall more active (* *p*<0.05; ** *p*<0.01).

In particular, *pdf-gal4/*P[UAS]^117^ (from now on referred to as *pdf*>P[UAS]^117^) exhibited an age-dependent decrease in the percentage of rhythmicity (Fig. 2 A–B and Table S2) resulting from an abnormal consolidation of the subjective day activity during constant conditions. This phenotype was not observed when analyzing in parallel a single copy of the *pdf*-*gal4* driver or the P[UAS]^117^ insertion in a heterozygous state (Fig. 2A–B). Overall activity, on the other hand, did not show any significant age-associated decline in *pdf-gal4*/P[UAS]^117^ and controls (Fig. 2C); moreover, no significant differences in overall activity were observed between aged *pdf-gal4*/P[UAS]^117^ and the *pdf-gal4* heterozygous control, suggesting that the progressive arrhythmicity characteristic of *pdf-gal4*/P[UAS]^117^ is not the result of a drastic reduction in total activity.

Our results suggest that GAL4 mediated alteration of the loci potentially affected by the P[UAS]^117^ insertion progressively impaired neuronal function giving rise to an age-dependent defective behavior.

### The P[UAS]^117^ insertion reduces the levels of endogenous *enabled*

**Figure 3 pone-0003332-g003:**
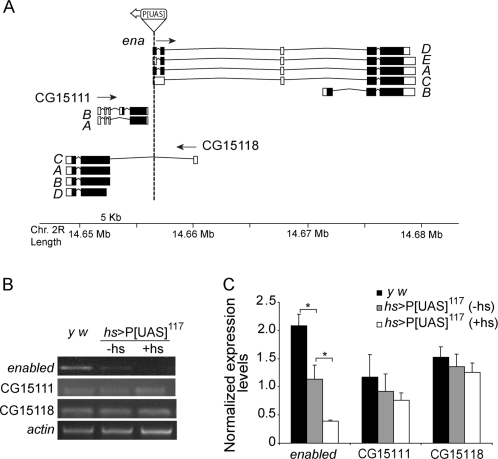
Plasmid rescue and RT-PCR analysis identifies *ena* as the affected locus. (A) Schematic diagram depicting the position of the P[UAS] transposon within the DNA region trapped by the insertion (to scale, in kb). The P[UAS]^117^ is located within the first exon of *ena* upstream of the ATG. It also landed within the first intron of CG15118 and near CG15111. Arrows indicate the direction of transcription for each gene. A–E refers to the different splicing variants in each locus. (B) To test the GAL4 mediated effect on each locus an ubiquitous heat shock (*hs*) GAL4 driver was employed. RT-PCR analysis was performed in *hs*>P[UAS]^117^ total larval RNA after a heat-shock stimulus (+hs). A non-pulsed control was used as baseline (-hs) along with a wild type control (*y w*). Semi quantitative RT-PCR products were analyzed on agarose gels stained with ethidium bromide (the image reflects *ena* levels at cycle 28). *actin* levels were compared for quality control of the independent RNA preparations and normalization. (C) The ratio between each independent gene to *actin* levels highlights significant changes only in the *ena* locus (* *p*<0.05). The insertion *per se* significantly reduces *ena* levels (* *p*<0.05). Statistical analysis included a Student t test. The experiment was repeated 3–4 times using independent RNA preparations.

Plasmid rescue analysis revealed that P[UAS]^117^ is inserted within the first exon of *enabled* (*ena*) upstream of the ATG, and thus it interrupts four out of the five splice variants predicted (Fig. 3A). The P element is located in reverse orientation with regard to transcription at this locus, potentially driving transcription of an antisense RNA in a GAL4-dependent manner. Such possibility is not unprecedented [18]. P[UAS]^117^ also interrupts the long splice variant of the predicted gene CG15118; it is located within its first intron, upstream of the exon containing the ATG in the same orientation. The transcriptional start sites of the three remaining splice variants lie nearly 5 kb downstream, and therefore it is unlikely that they will be affected. Within this region there is a third predicted gene (CG15111) that runs in the opposite orientation to P[UAS]^117^ but it is not physically interrupted by it.

To assess the impact of GAL4 directed expression on the potentially affected loci total RNA was extracted from *hs*>P[UAS]^117^ heat-shocked larvae and non-pulsed controls along with a wild type strain (*y w*). Semi quantitative RT-PCR analysis with primers directed to a region present in all splice variants for each gene was carried out and normalized expression levels are shown (Fig. 3B, C). P[UAS]^117^ appears to strongly and specifically affect *ena* levels, while little or no change was observed for CG15111 and CG15118. The insertion *per se* reduced *ena* levels to about 50 percent of wild type controls (*y w*). Interestingly, heat-shocked flies showed about one third of *ena* levels compared to non-pulsed controls thus confirming that GAL4-driven expression is triggering the decrease of endogenous *ena* levels.

We therefore renamed P[UAS]^117^ as *ena^reverse(rev)^* to reflect that GAL4 mediated expression results in downregulation of the *ena* locus; when crossed to a GAL4 source such scenario gives rise to a tissue-specific hypomorphic mutation.

### Progressive behavioral phenotypes are the result of reduced *ena* levels

**Figure 4 pone-0003332-g004:**
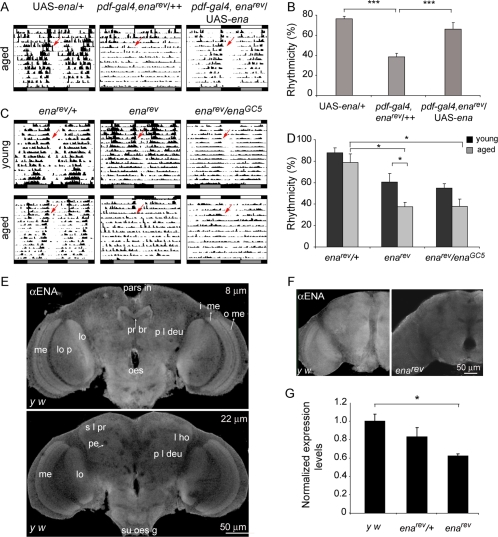
Age associated arrhythmicity is dependent on *ena* function. (A) Rescue of ENA function on the PDF circuit restores behavioral rhythmicity in aged flies. Recombinant *pdf-gal4*, *ena^rev^* carrying one copy of UAS-*ena* were indistinguishable from control UAS-*ena*. Representative double plotted actograms for aged (24–28d) flies are shown. Overexpression of ENA in young flies do not affect locomotor activity rhythms (data not shown). (B) The percentage of rhythmicity for aged flies for each strain is shown. *pdf-gal4*, *ena^rev^*/++ is significantly different from both UAS-*ena*/+ (control) and *pdf-gal4*, *ena^rev^*/UAS-*ena* (*** *p*<0.001). (C) Representative actograms of young (3d) and aged (21d) flies carrying one or two copies of *ena^rev^*, along with the transheterozygotes *ena^rev^*/*ena^GC5^*. Both homozygous *ena^rev^* and *ena^rev^*/*ena^GC5^* exhibit a decline on rhythm strength. (D) Summary of behavioral data for flies of the indicated genotypes. Control *ena^rev^/+* flies stay rhythmic throughout lifespan. Aged *ena^rev^* is significantly different from its younger counterpart (* *p*<0.05). Both aged *ena^rev^* and *ena^rev^*/*ena^GC5^* are different from old *ena^rev^/+* (* *p*<0.05). Experiments summarized in B and D were repeated at least 3 times. (E) Whole mount brain immunofluorescense of 10 day-old adult *y w* flies were stained with a specific antibody against ENA. A general staining of neuropils in the adult brain was observed. Single confocal planes (2 µm thick) are shown at two depths (8 and 22 µm) to highlight different brain areas. Some of the neuropils labeled with ENA are the outer (o me) and inner medulla (i me), lobula (lo) and lobula plate (lo p) within the optic lobe, the protocerebral bridge (pr br) in the central complex as well as other regions in the protocerebrum such as the lateral horn (l ho). Other structures, as the protolateral deutocerebrum (p l deu), superior lateral protocerebrum (s l pr), the peduncles of the mushroom body (pe), pars intercerebralis (pars in), suboesophageal ganglion (su oes g) and oesophagus (oes) are also shown in the figure. (F) *ena* levels are reduced in *ena^rev^* mutants compared to the control *y w*. Images were taken with the same confocal settings for direct comparison; projections of 2.3 µm depth are shown. ENA immunohistochemistry was repeated at least three times. (G) RT-PCR analysis was performed in adult *ena^rev^*, *ena^rev^/+* and control total RNA. The ratio between *ena* and *actin* expression levels for each genotype is shown. Quantification of RNA levels showed significant changes in *ena^rev^* homozygous (* *p*<0.05) whereas a minor (non significant) decrease was seen in *ena^rev^/+* heterozygous when compared to the *y w* control line. The experiment was repeated three times employing independent RNA preparations.

To determine whether *ena* down-regulation by itself could be responsible for the progressive arrhythmicity two complementary approaches were carried out. To assess whether increasing *ena* expression within the GAL4-mediated hypomorph is sufficient to rescue wild type behavior, a copy of UAS-*ena* was introduced in *pdf>ena^rev^*. Restoring ENA levels reduced the arrhythmicity of aged *pdf>ena^rev^* which became undistinguishable from control flies (UAS-*ena*/+, Fig. 4A–B).

On the other hand, the homozygous *ena^rev^* insertion *per se* showed a progressive decline in the degree of rhythmicity in older flies (Fig. 4C, D), likely due to a reduction in *ena* levels (Fig. 4F). To test whether other strategies to decrease *ena* levels could also give rise to arrhythmic behavior we tested *ena^rev^* effect on locomotor activity in the context of a well characterized null mutant (*ena^GC5^*) [28]. Transheterozygotes *ena^rev^*/*ena^GC5^* should recreate the defects observed in homozygous *ena^rev^* flies if reduced ENA levels were the sole responsible for the phenotype. Figure 4C shows progressive arrhythmic behavior for *ena^rev^*/*ena^GC5^* transheterozygotes, phenocopying homozygous *ena^rev^*, thus ruling out the contribution of unrelated loci potentially affected by the P-element insertion in *ena^rev^*. Interestingly, both *ena^rev^* and *ena^rev^*/*ena^GC5^* showed signs of deconsolidated activity as young adults. Neither *ena^GC5^* nor *ena^rev^* showed any defects when a single copy was examined (Fig. 4C–D and Table S2).

To rule out a potential contribution of altered CG15118 expression (undetectable by RT-PCR, Fig. 3C) to the arrhythmic phenotype observed in homozygous *ena^rev^*, we tested *ena^rev^* in the context of a different P-element insertion (18105) that only reduces CG15118 levels (confirmed by RT-PCR analysis, not shown). As shown in Table S2, aged 18105/*ena^rev^* individuals were highly rhythmic, making the contribution of this locus to the behavioral phenotype rather unlikely.

Altogether this data supports the notion that progressive arrhythmicity specifically derives from down-regulated *ena* levels.

### *ena* is expressed in the adult brain neuropil

*enabled* encodes a protein that links signaling pathways to the remodeling of actin cytoskeleton, and therefore is crucial for a variety of cellular process including morphogenesis, cell migration and adhesion [29]. As such it has been implicated in axon pathfinding during nervous system development [28]. However, a role for ENA in the adult brain has never been addressed.

To determine whether *ena* is expressed in the adult brain, we carried out immunofluorescence on whole mount brains employing an anti-ENA specific monoclonal antibody [30]. ENA exhibited a homogeneous signal that was localized in several neuropils, reminiscent of those expressing synaptotagmin [31]. Primary sensory centers like the visual lamina (lamina, medulla, lobula and lobula plate in the optic lobe) as well as some central brain regions including the central complex were stained (Fig. 4E). The lateral horn, the superior lateral protocerebrum, and the lateral deutocerebrum also showed ENA immunoreactivity.

Immunohistochemical analysis revealed that ENA expression was strongly reduced in homozygous *ena^rev^* adults (Fig. 4F). RT-PCR analysis indicated that homozygous *ena^rev^* showed a significant reduction in *ena* expression, whereas a single copy of P[UAS]^117^ (as in *ena^rev^/+*) resulted in a subtle decline in *ena* levels, consistent with its lack of effect on the behavioral paradigm (Figs. 4G and 2A–B).

Detection of ENA in the adult brain indicates that this protein is present throughout the life of the organism, and thus its down-regulation could be triggering accumulative defects that in time result in behavioral impairment.

### The adult optic lobe is particularly vulnerable to *ena* miss-regulation

**Figure 5 pone-0003332-g005:**
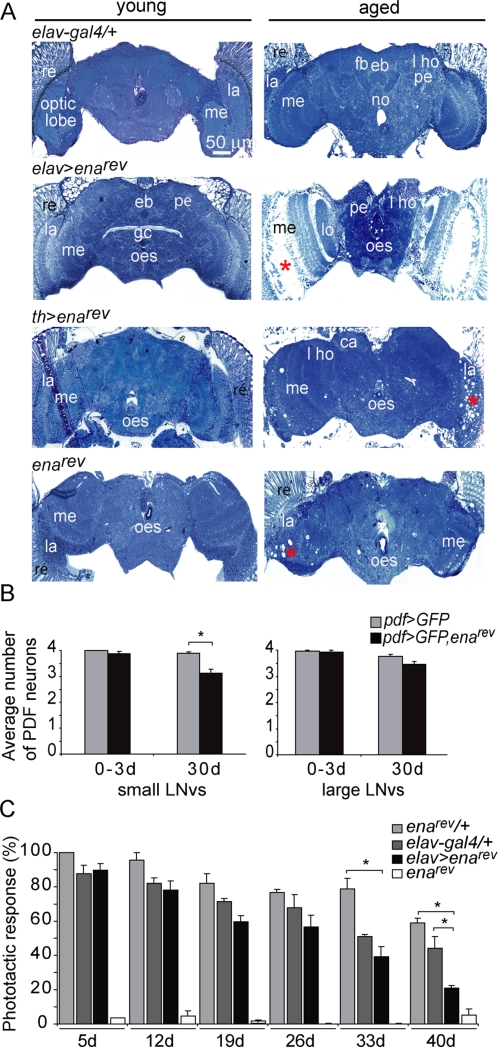
(A) Decreased ENA levels triggers progressive degeneration in the optic lobe. Frontal adult head semi-thin sections (1 µm thick) were stained with methylene blue and examined by light microscopy. Representative sections are included. Two different drivers were employed in order to reduce ENA levels; *elav*-*gal4*, a panneuronal driver, and *th-gal4*, driving GAL4 expression specifically in the dopaminergic neurons. Young (0–3 days) and old flies (30 days) were analyzed for each genotype. Reduction of ENA levels both panneurally and in the dopaminergic system causes degeneration in the same areas of the brain (indicated by a red asterisc within the affected region). *elav>ena^rev^* flies show age dependent vacuolization in the medulla and the lamina within the optic lobe while the nervous system of the control line (*elav*-*gal4*/+) is well preserved throughout the time evaluated. Reduced ENA levels exclusively in dopaminergic neurons (*th>ena^rev^*) also leads to vacuolization in the optic lobe in aged flies, although to a lower extent. *ena^rev^* shows specific degeneration within the optic lobe, characterized by the occurrence of vacuoles in the medulla and lamina mainly in older flies. Four to ten heads were analyzed per genotype in 2–5 different experiments. Different regions in the adult brain are indicated as follows: medulla (me), retina (re), lamina (la), lateral horn (l ho), peduncles of the mushroom body (pe), fan-shaped body (fb), ellipsoid body (eb), calix (ca), lobula (lo), suboesophageal ganglion (su oes g) and oesophagus (oes), giant commissure (gc), nodules (no). (B) Progressive expression of *ena^rev^* reduces the number of PDF neurons. *pdf*-*gal4*>*GFP* and *pdf*-*gal4*>*GFP*; *ena^rev^* adult brains were dissected and the number of GFP positive small (s-LNvs) and large (l-LNvs) neurons per hemisphere was counted in young (0–3 day old) and aged (30 day old) individuals. Statistical analysis included pairwise comparisons employing Student t test. The s-LNvs showed a small but significant decrease in the number of PDF positive neurons (* *p*<0.05). No differences in the large LNv cluster were observed. (C) Vacuolization within the optic lobe progressively impairs a behavioral response in *elav>ena^rev^*. In a longitudinal assay the phototactic response of flies of the indicated genotypes was examined. The performance in this paradigm decreased as the flies aged for all genotypes. As anticipated from the estructural defect *elav>ena^rev^* flies performed progressively worse, becoming significantly different from both genetic controls by the end of the experiment (* *p*<0.05). Homozygous *ena^rev^* flies did not show any clear response in any of the paradigms tested (see also Fig. S2).

To address whether down-regulated ENA function could lead to degeneration within the brain we employed the panneuronal driver *elav* [32]. Semi-thin frontal sections stained with methylene blue were analyzed in young and aged flies. Mutant brains (*elav>ena^rev^*) from old flies displayed significant cortex and neuropil vacuolization (Fig. 5A). This phenomenon was not apparent in parental strains (*elav-gal4*/+ and heterozygous *ena^rev^*) or in young *elav>ena^rev^* flies, revealing an age dependence of the neuropathological phenotype.

Vacuolization in *elav>ena^rev^* brains was not widespread; instead, specific regions such as the medulla and the lamina in the optic lobe were particularly vulnerable to deregulated ENA, which is also supported by the observations made in *ena^rev^* homozygous mutants (Fig. 5A). To rule out potential artifacts due to region-specific expression levels associated to the *elav-gal4* driver, we targeted ENA misexpression to dopaminergic neurons employing *th-gal4* [33]. Interestingly, even though dopaminergic neurons are scattered throughout the adult brain, in *th>ena^rev^* only the optic lobe showed clear vacuolization, although to a lower extent than *elav>ena^rev^* (Fig. 5A). Moreover, *ena* misexpression in regions not including the optic lobe did not trigger any sign of neuronal death (such as the central brain, see the *C309*>*ena^rev^* [34] example shown in Fig. S1). The fact that the somatas of the small LNvs are located within a region highly vulnerable to *ena* misregulation likely accounts for the behavioral phenotype; in fact the total number of PDF reactive neurons is reduced in 3 week old *pdf>ena^rev^* flies (Fig. 5B).

Given the extent of vacuolization observed in the optic lobe, a neuropil involved in visual processing, we investigated whether the natural response towards light (the phototactic response [35]) was impaired in *elav>ena^rev^* individuals. Eventhough most genotypes displayed a reduced phototatic response as the flies aged, *elav>ena^rev^* flies showed a marked decrease of its performance in this behavioral task after 4 weeks of age (Fig. 5C). Not surprisingly given the nature of the mutation and the number of cell types that could be defective in this hypomorphic mutant, homozygous *ena^rev^* flies barely moved towards light; most likely this lack of response reflects an impaired ability to react to stimuli, since homozygous *ena^rev^* also performed poorly in a climbing assay (Fig. S2).

Taken together, our observations demonstrate that reduced *ena* levels cause neuronal dysfunction in specific areas of the adult brain, leading to progressive behavioral abnormalities and neuronal death.

### Reduced *ena* levels triggers axonal clogging

**Figure 6 pone-0003332-g006:**
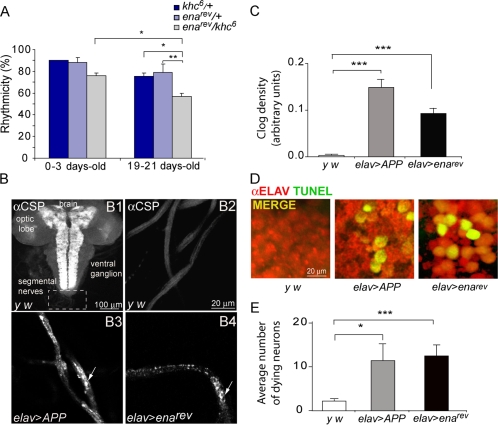
Reduced *enabled* levels triggers axonal swellings. (A) Genetic interactions between *ena^rev^* and *khc^6^* suggest that axon transport defects could be underlying the progressive loss of rhythmicity. The percentage of rhythmic flies for each strain is shown. Transheterozygote *ena^rev^/ khc^6^* flies are significantly different from its younger counterpart (* *p*<0,05 ) and from the heterozygote aged controls (** *p*<0,01 and * *p*<0,05 when compared to *ena^rev^*/+ and *khc^6^/+*, respectively). Experiments were repeated at least three times. Additional details are included Table S2. (B) Third-instar larval segmental nerves were stained against CSP, a synaptic vesicle protein. (B1) The whole larval preparation is shown. The dashed box corresponds to the region shown in B2–B4. (B2) Segmental nerves from control larvae exhibit relatively uniform CSP staining. (B3–B4) However, large immunoreactive CSP clusters (arrows) are observed in the segmental nerves of the positive control *elav>APP* (B3) as well as *elav>ena^rev^* (B4) larvae. (C) Quantitative analysis on larval segmental nerves was performed by measuring clog density. Fourteen to thirty five brains were examined. *elav>ena^rev^* was significantly different from the wild type control (* *p*<0.05), similarly to what was seen for *elav>APP* (*** *p*<0.001). (D) Representative images of TUNEL staining on the indicated genotypes. (E) Quantitative analysis of TUNEL staining showing the extent of neuronal death in *elav>ena^rev^* and a positive control, both significantly different from a wild type control (* *p*<0.05, *** *p*<0.001).

Fast-axonal transport cargoes, such as vesicle-associated synaptic terminal proteins and mitochondria, can accumulate in axonal swellings derived from mutation of kinesin 1 or dynein [36]–[39]. ENA has been found to directly interact with kinesin heavy chain (Khc), a molecular motor involved in fast axonal transport [40]. To examine the possibility that defective axonal transport could contribute to the progressive behavioral phenotype, genetic interactions between a *khc* mutant (*khc^6^/+*) and heterozygous *ena^rev^* were examined. Interestingly, transheterozygous *ena^rev^/khc^6^* flies displayed progressive loss of rhythmicity (Fig. 6A), suggesting an impairment at this level. To examine whether ENA down-regulation could give rise to abnormal cargo accumulation, the localization of the synaptic vesicle protein CSP in the larval segmental nerves (Fig. 6B) was examined [25], [41]. Axonal clogs are aggregates of membrane bound cargoes and can be a consequence of defective axonal transport [36]. As a positive control APP was overexpressed (*elav>APP*), a manipulation that has already been demonstrated to induce axonal clogging [25], [42]. Consistent with this notion, the segmental nerves in *elav*>*APP* flies displayed conspicuous clusters of the presynaptic protein CSP (Fig. 6B3), which were absent in wild type controls (Fig. 6B2). Strikingly, reduced ENA levels in *elav>ena^rev^* also resulted in the development of axonal clogs (Fig. 6B4), suggesting impairment at this level. The density of axonal clogs was then measured; *elav>ena^rev^* flies were significantly different from wild type controls similarly to what was seen for *elav>APP* (Fig. 6C). Comparable results were obtained when the localization of the synaptic protein SYT was analyzed (data not shown).

Earlier work has shown that APP misregulation leads to apoptosis [25]. To investigate whether reduced *ena* levels could also trigger this mechanism TUNEL staining was performed on larval brains (Fig. 6D–E). Strikingly, increased cell death correlated with continuous down-regulation of *ena* levels, suggesting that the abnormal organelle accumulations observed in the *elav>ena^rev^* mutant results in apoptotic cell death.

Taken together these results are consistent with the notion that reduced *ena* levels causes transport dysfunction of certain specific cargoes thus contributing to the degenerative phenotypes.

### Constant *ena* down-regulation is associated with progressive apoptosis

**Figure 7:**
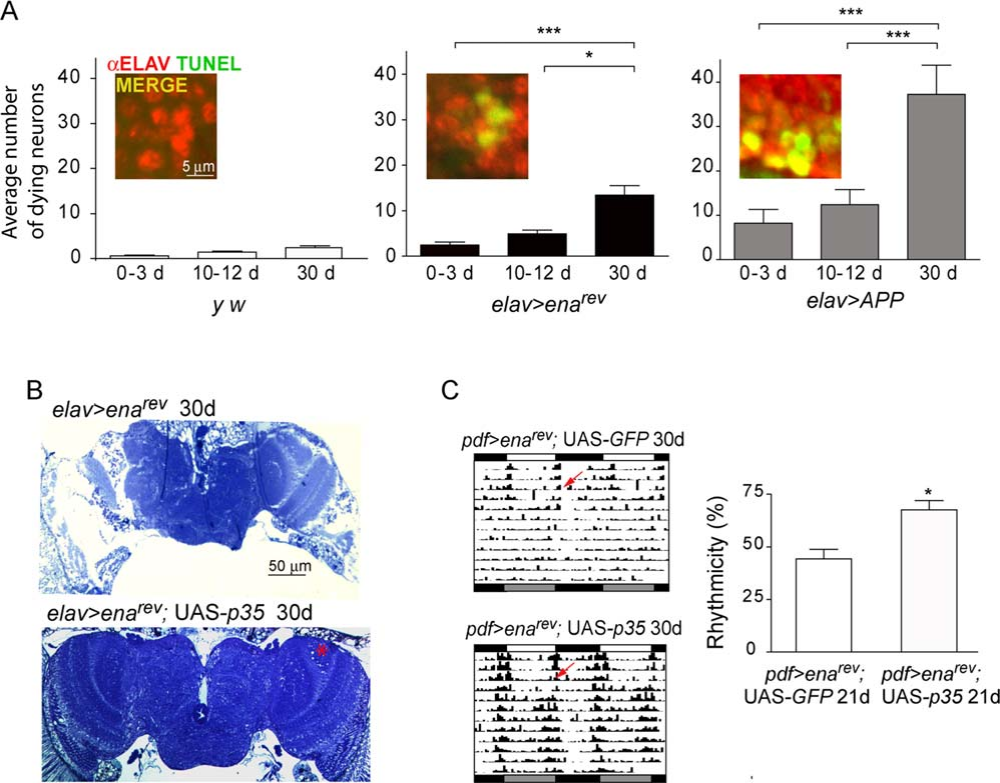
*ena* down-regulation correlates with progressive cell death. (A) Quantitative analysis of apoptotic cell death in adult brains of increasing age as indicated in the figure. A representative image of the extent of cell death in 30 day-old flies of the indicated genotypes is shown in the inset (upper corner, left). Twenty to fifty brain hemispheres were analyzed in each time point with exception of 30 day-old *elav>APP* (n = 11). The degree of apoptosis in the affected individuals is significantly different from a wild type control (* *p*<0.05, ** *p*<0.001). (B–C) Degenerative phenotypes associated to *ena^rev^* are rescued by *p35* expression. (B) Frontal sections at approximately the same depth of aged control and *elav>ena^rev^*, *p35* brains highlight the extent of the morphological rescue. The asterisk denotes a region where small vacuoles can still be found in one of the few brains in which the rescue was not complete. (C) Functional rescue of *ena-* derived behavioral phenotypes. Representative actograms of old *pdf>ena^rev^*, *p35* and control lines are included (left panel). The percentage of rhythmic individuals is also shown (right panel, * *p*<0.05).

Reduced *ena* levels correlated with positive TUNEL staining in the larval brain; however young adult flies did not develop behavioral or anatomical defects. During metamorphosis the development of novel neuronal clusters and connections could generate a new architecture susceptible to *ena* down-regulation, which only in time would display such defects. In control brains a minimum level of TUNEL staining was observed scattered throughout the brain, which did not significantly increase in older flies (Fig. 7A). However, when *elav>ena^rev^* brains were stained, an increasing number of apoptotic neurons in the optic lobe was observed, albeit to a lower level than after APP overexpression. This data is consistent with a scenario in which constantly reduced *ena* levels lead to neuronal dysfunction and eventually trigger apoptosis, in time affecting a larger and differentially susceptible neuronal population, thus accounting for the progressive behavioral and anatomical defects.

To evaluate whether the extensive vacuolization observed in aged individuals derived solely from apoptotic cell death, a single copy of *p35*, a general caspase inhibitor [43], was introduced in *elav>ena^rev^*. Remarkably, most of the aged *elav>ena^rev^*, *p35* brains displayed no vacuolization, while only a few showed small vacuoles localized to the most susceptible regions (Fig. 7B). The rescue of arrhythmicity observed in *pdf>ena^rev^*, *p35* flies (Fig. 7C) underscores that regardless of additional mechanisms underlying ENA-mediated neurodegeneration, programmed cell death is an important effector.

## Discussion

### An adult-based automated behavioral screen identifies genes potentially involved in neurodegeneration

Despite being an area of intense research, the complexity of the underlying phenomena is such that a clear picture of the variety of molecular mechanisms triggering neurodegeneration remains elusive. We present a novel forward genetic screen based on misexpression within a dispensable circuit [20] to identify genes involved in neurodegeneration. The screen relies on the direct comparison of an automated behavioral output at two times during adulthood. We focused our screen on mutations specifically causing late onset defects, since this is a typical characteristic of neurodegenerative diseases [44]. We envision that subtle changes in the expression of genes playing essential roles in neuronal homeostasis and physiology will in time impact behavior, and more importantly, they could account for several common features displayed in degenerative pathologies of the nervous system, such as late onset, protein aggregation, or specific vulnerability of particular brain regions. Among the interesting candidates is a P[UAS] that landed in CG3875, a predicted gene encoding a transcription factor potentially linked to apoptosis. The fact that misexpression of a putative pro-apoptotic gene results in progressive loss of rhythmic behavior underscores the validity of our strategy.

Although the anticipated outcome of a misexpression screen is increased levels of the trapped gene, we focused our attention in P[UAS]^117^, a GAL4-mediated tissue-specific hypomorph. Previously described loss-of-function mutations in *enabled* are embryo lethal, and since one copy of the null alleles display no clear defects, it is highly unlikely that this gene would have been associated to a progressive adult phenotype in the course of a traditional loss-of-function screen.

*enabled* is a member of the ENA/VASP family involved in actin cytoskeleton remodeling through the modulation of actin filament length, branching and bundle formation. ENA has been studied during early development, and was described as an important regulator of growth cone dynamics, cell migration and adhesion, where it promotes the formation of long unbranched actin filaments (as a review [45]).

We have shown that constantly reduced *ena* levels throughout the lifespan are associated with progressive behavioral defects and cell death, which become evident only in aged individuals; interestingly, certain brain regions such as the optic lobe are particularly vulnerable to *ena* down-regulation (Figs. 3 and 5; also see below), thus recreating several hallmarks of neurodegenerative diseases (late onset, progression, distinct vulnerability). These findings are reminiscent of what was reported by Kretzschmar and colleagues when characterizing a hypomorph mutant of *futsch*, a MAP1B related protein [46]. They found defective mitochondrial transport in cultured neurons from pupae, preceding the behavioral abnormalities and neuronal death, which became evident later in the adult life. In support to the notion that altered ENA levels lead to neurodegeneration, the worm ortholog of *ena* has recently been shown to modulate neurotoxicity derived from *tau* over-expression [47].

### Down-regulation of *ena* results in cargo accumulation

Given the variety of cellular processes in which ENA is involved [29], [48], it is not unlikely that the neurodegeneration exhibited in adult flies would derive from a number of affected phenomena. Recently, Feany and colleagues showed that abnormal bundling of actin filaments accounted for the neurodegeneration observed in a fly and mouse models of tauopathy [49]. However, no gross changes in F-actin levels or distribution were found in the brains of adult *ena^rev^*. Along this line, genetic interactions with *actin* mutants in the context of heterozygous *ena^rev^* did not exhibit behavioral defects (data not shown), precluding the establishment of a direct association between these two components in our model of neurodegeneration.

Neurons are highly polarized cells where most metabolic processes take place within the soma, also the primary site for the novo protein synthesis later feeding the dendritic and axonal processes; as such they are highly dependent on an efficient cellular transport supported by the interaction between microtubules, microfilaments and intermediate filaments. Transport deficits have been reported in neurodegenerative diseases such as those caused by polyglutamine expansion in HD [50], [51], as well as mutations associated with dysfunction of cytoskeletal components that influence vesicular biogenesis, vesicle/organelle trafficking and synaptic signaling (as an example [49]). Moreover, neuropathies have been associated to patients with missense mutations in *dynactin* or *kinesin* [52], reinforcing the connection between transport and neurodegeneration. Our data is consistent with the notion that reduced ENA levels impair the efficient flow of crucial cargoes to the synaptic terminals altering proper neuronal function, in turn leading to cell death (Figs. 5–7); a fast axonal anterograde transport failure, likely mediated by ENA-kinesin 1 interactions [40], could mediate this effect. In fact in fly models of AD, APP and APPL overexpression have been associated to transport defects [53], and were proposed to correlate to impaired synaptic plasticity [42]. Interestingly, APP overexpression leads to progressive defects in our behavioral paradigm (Fig. 1E, F), and paralleled some of the abnormalities observed in *ena^rev^* mutants (Figs. 1, 6 and 7), suggesting that similar cellular mechanisms could be affected in our model of neurodegeneration.

Alternatively, *ena* down-regulation could interfere with the dynamic interaction between the actin cytoskeleton and the microtubule network, which has been shown to share certain small molecule regulators (for references see [54]). In fact, it has been shown that loss of ENA/VASP function precludes neuritogenesis, which could result from an impaired interaction between these two systems [55]. Genetic interactions between *ena^rev^* and several anterograde and retrograde motor proteins lend support to this possibility (Fig. 6A and data not shown). However, since only certain neuronal populations appear vulnerable to *ena* down-regulation we favor the idea that specific cargoes are affected as opposed to a general transport failure, highlighting the exquisite regulation over motor proteins and their cargos.

### *ena* down-regulation leads to apoptotic cell death

Aged *elav>ena^rev^* individuals display extensive vacuolization in specific areas of the adult brain which correlated with a decreased response in a phototaxis assay, likely as a result of programmed cell death (Figs. 5 and 7), which was largely rescued when a general caspase inhibitor was introduced in the *ena*-defective background. However, a minor degree of vacuolization was still observed in the most affected areas. In agreement with these observations, *pdf>ena^rev^*, *p35* flies displayed no behavioral abnormalities as flies aged, supporting a functional rescue of the *ena*-mediated neuronal dysfunction. Increasing evidence suggests that apoptotic biochemical cascades are involved in the dysfunction and death of neurons in neurodegenerative disorders such as AD, PD, and HD. In this regard, Rohn and colleagues have shown that overexpression of the anti apoptotic protein Bcl-2 in the context of a triple transgenic AD mouse model results in lack of pathology, highlighting the contribution of caspases in disease progression [56].

Taken together our results underscore that ENA not only is required during the establishment of the embryonic nervous system but also in maintaining the cellular homeostasis of the adult nervous system. Given the increasing interest in understanding neurological disorders and the conservation of ENA function in higher eukaryotes, progress in this model system will have a broad impact ultimately assisting in the dissection of pathways relevant to neurodegeneration in the mammalian brain.

## Materials and Methods

### Strains and fly rearing

*y w* and Canton-S flies were employed as wild type controls. The following fly lines were provided by the Bloomington Stock Center: *y w* (**1495**); C S (**1**); *w* ^*^; *ninaE*.GMR-*gal4* (**1104**); *Adh^fn6^ pr^1^ cn^1^ ena^GC5^*/CyO (**8570**); P[GawB]*elav^C155^* (**458**); P[GAL4-Hsp70.sev]2 /CyO; ry^*^(**2023**); *y w*; P[Pdf-GAL4.P2.4] (**6900**) and *w* ^*^; P[GawB]c309 (**6906**); w*; P[UAS-APP695-N-myc]TW6 (**6700**); y1 w*; P[UAS-*ena*.AD]3 (**9139**); *w* ^1118^; PBac[RB]CG15118^e03002^CG15111^e03002^ (**18105**) and y1 w*; P{UAS-mCD8::GFP.L}LL5 (**5137**). The fly lines *th-gal4* and w;c- *khc6*/Cyo were kindly provided by Scott Waddell (University of Massachusetts Medical School) and William Saxton (Department of Biology, Indiana University, Bloomington), respectively. The recombinant *pdf*-*gal4*, *ena^rev^* was generated in our laboratory.

*Drosophila* cultures were maintained on a 12 hr light/dark (LD) cycle on standard corn meal yeast agar medium at 25°C in an environmental chamber. Ageing flies were transferred into fresh vials at least once a week throughout the experiment.

### Misexpression screen

New insertions of the P[UAS] were generated mobilizing the EP55 line described in [15] via the transient introduction of the transposase (Δ2–3) [57]. Subsequent mobilizations were achieved using an insertion in the 4^th^ chromosome (P[UAS]^8^). Independent lines were mapped and stocks were kept for further analysis. *The pdf*-*gal4* line [24] was crossed to the entire P[UAS] collection to deregulate the endogenous trapped genes exclusively in the circadian pacemaker circuit. Young (0–3 days) and aged (at least 21 days) flies from each cross were tested on locomotor activity and the percentage of rhythmic flies was compared.

### Behavioral analyses

Fly activity was monitored under LD conditions for 4 days when flies were released into DD at least for one week employing commercially available activity monitors (TriKinetics, Waltham, MA). Activity of individual young (0–3) and aged (21 day-old) flies was examined. Period and rhythmicity were estimated using the ClockLab software (Actimetrics, Evanston, IL) from data collected in constant darkness. Flies with a single peak over the significance line in a Chi-Squared analysis were scored as rhythmic, which was confirmed by visual inspection of the actograms. The FFT parameter represents the strength of rhythmicity. Flies classified as weakly rhythmic were not taken into account for average period calculations [58]. Total activity levels were determined as total counts per day displayed for each fly. Data shown in figures 1, 2, 4 and 7 was obtained from at least three independent experiments.

### Phototaxis assays

Phototaxis assays were performed essentially as described [35] with modifications. A new horizontal device was developed that allows the “collecting tubes” to slide through the one containing the flies at the beginning of the experiment, which is always kept in the same position. For the longitudinal assays about 100 males of each genotype were kept in standard food and transferred to fresh vials every 3–4 days. Flies were tested once a week at the same time. Twenty four hours before testing 30 flies per genotype were randomly selected under CO2 and placed in a fresh vial with standard food. At least 15 min before testing flies were transferred to darkness; further manipulations were performed under safe red light. Flies were moved to a glass ¨tesẗ tube (10 cm long, 1 cm wide). Five clean ¨collecting̈ tubes were placed opposite to the original ¨tesẗ one containing the flies. The white cold light source (100 watts, Leica CLS) was initially placed right behind the collecting tube 1, and kept in line with the test tube throughout the experiment. Each collecting tube was allowed to connect sequentially with the test tube for 1 min. Thus, flies that quickly moved towards the light source were contained in the first collecting tube and those that did not respond at all stayed within the test tube. The number of flies in each (collecting and test) tube was counted, and the percentage of flies that had a positive phototactic response (defined as those that moved towards the light in the first 2 min of the test) is shown in Fig. 5C. Statistical analysis included a two way ANOVA followed by Tukey pairwise comparisons (Infostat group, UNC, Argentina).

### Climbing assays

Climbing assays were performed essentially as described [59]. In the longitudinal assays about 100 males of each genotype were kept in standard food and transferred to fresh vials every 3–4 days. Flies were tested once a week at the same time. The day before testing 10 flies per genotype were randomly selected, placed in fresh food, and transferred to the empty vials of the RING apparatus 20–30 min prior to the assay. Each assay begins with a gentle tapping to bring all the flies down; the ability of each individual to exhibit a coordinated response is evaluated after 5 seconds through a digital camera. Data acquisition and analysis was performed as described [59].

### RT-PCR

To identify the ORFs affected by GAL4 mediated expression *hs*-*gal4*/ P[UAS]^117^ larvae were heat-shocked at 37°C for 30 minutes in a water bath and allowed to recover at 25°C for 2 h before processing. This procedure was repeated twice. Non-pulsed controls and a wild type strain (*y w*) were used for comparison. Total RNA was isolated employing Trizol (Invitrogen). Reverse transcription was performed using the SuperScript first-strand synthesis system (Invitrogen) according to the instructions of the manufacturer. Semi quantitative PCR analysis was performed using the following primers: *enaFw* 5′-CCCTTGAAAAGCCCAAACAC-3′ and *enaRv* 5′-CCGGGCCTGATTGTACTTC-3′; *15118Fw* 5′-AGGAAGCTTCCAACGCTGGAGT-3′ and *15118Rv* *5′*-CAAGAGGAATTTGCCGACGG-3′; *15111Fw* 5′-TGTTCATCTCTGGCTGTCATCG-3′ and *15111Rv* 5′-CCT GACGTGATCCTTTACGGT-3′. *actinFw* 5′- GAGCGCGGTTACAGCTTCAC-3′; *actinRv* *5′*- ACTCTTGCTTCGAGATCCACA-3′. The products obtained at different cycles during the progression of the reaction (25, 28, 31 and 35) were then analyzed on agarose gels stained with ethidium bromide. Quantitation was performed on products obtained in cycle 28 employing the Image J software. RT-PCR analysis was also performed in adult *ena^rev^*, *ena^rev^/+ and y w* total RNA. Quantitation of the expression levels of each gene normalized to *actin* levels was performed for each genotype. Experiments were repeated 3–4 times on independent RNA preparations.

### Whole brain Immunohistochemistry

Adult brains were dissected while flies were pinned down to a sylgard dish in phosphate-buffered saline (PBS), and then fixed in 4% paraformaldehyde in PB (100 mM KH_2_PO_4_,/Na_2_HPO_4_) between 30 minutes and 1 hour at room temperature. The excess fixative was removed by rinsing three times in PT (PBS plus 0,1% Triton X-100). Brains were then blocked in 7% goat serum in PT for 2 hr at room temperature. After the blocking step tissue was incubated with the primary antibody for 72 h at 4°C, and then washed for three times with PT for 20 minutes prior to the addition of the secondary antibody. After a 2 h incubation step, brains were washed for three times in PT and mounted in 80% glycerol (in PT).

The primary antibodies used were mouse anti-ENA (1/5, Developmental Studies Hybridoma Bank) or chicken anti-GFP (1/500, Upstate technologies). The secondary antibodies used were donkey Cy3-conjugated anti-mouse, Cy2-conjugated anti-chicken (1/250, Jackson ImmunoResearch) and Alexa 594 anti-mouse (1/250, Invitrogen). Detection of ENA in the adult brain was repeated at least three times examining 8–10 brains in each experiment. To compare ENA levels between wild type and mutant brains confocal fluorescence images were taken under the same conditions. A confocal Zeiss LSM510 microscope was used to image whole adult brains and larval preparations.

TUNEL staining on non fixed larval and adult brains was performed according to the manufacturer's recommendations (Apoptag Plus Fluorescent Kit, Millipore). Co-localization with ELAV (a neuronal marker) was used as counterstain.

### Semi-thin sections

Frontal adult head semi-thin sections (1 µm thick) were stained with methylene blue. Heads were fixed with 3% glutaraldehyde in PBS for 2 h at room temperature, treated for 1–2 h in 1% osmium, dehydrated through several ethanol-steps and embedded in Spurr's epoxy resin. Four to ten heads from 0–3 or 30 day-old flies were analyzed per genotype in different trials occasions. Intermediate ages were examined for certain genotypes. Sections were visualized in a BX-60 Olympus microscope and photographed with a CoolSnap Pro digital camera.

### Larval preparation, immunohistochemistry, and quantification

Larval brains from third-instar larvae were first removed preserving the associated segmental nerves in PBS, fixed in 4% formaldehyde PBS for 1 h at 25°C and then rinsed in PT. Samples were blocked in 7% goat serum in PT for 40 min at room temperature and then incubated with the primary antibody for 48 h at 4°C. Brains were then washed three times with PT for 40 min, followed by a 2 h incubation with the secondary antibody. After staining, brains were washed three times with PT and mounted in 80% glycerol (in PT). Anti-REPO was used as a counter stain. Primary antibodies used were anti-CSP, SYT and REPO at a final concentration of 1/5 (all of them obtained from the DSHB). Secondary antibodies were Cy2-conjugated goat anti-mouse IgG1 and Cy5 conjugated goat anti-mouse IgG2b (1/250, Jackson ImmunoResearch).

Quantitative analysis on larval segmental nerves was performed essentially as described in [25] with minor modifications. Three confocal optical images each 1.3 µm apart in the z axis were collected from 2–3 different nerves per larva. Each nerve was analyzed over a length of 200 µm by measuring the area spanned by the clogs compared to the total area, employing the analysis package included in Image Pro Plus (Media Cybernetics, Silver Springs, MD). For quantitation purposes each genotype was represented by four to five independent experiments comprising a total of twelve to sixteen different larvae.

### Statistical analysis

Statistical analysis was performed employing the Prism Graphpad 4.0 software package (2003) unless otherwise noted (Figs. 5C and S2). ANOVA and pairwise comparisons employing Student's *t* test with the Bonferroni correction for multiple comparisons were used in figures 1–7 (with exception of Figs. 5C and S2 as indicated in the corresponding figure legend).

## Supporting Information

Figure S1 Frontal adult head semi-thin sections (1 µm thick) were stained with methylene blue and examined by light microscopy. Representative sections are included. Young (0–3 day old) and old (30 day old) flies were analyzed for each genotype. Heterozygous *ena* ^rev^ flies show no signs of degeneration throughout adulthood. The *C309* driver was employed to reduce ENA levels in the central brain (indicated by the dashed line) but not in the optic lobe. Neither young nor aged *C309>ena^rev^* flies show any sign of neurodegeneration. The control insertion line P[UAS]^218^ displaying high levels of rhythmicity at older stages was also evaluated employing the panneural *elav* driver. No evidence of vacuolization was observed in young or aged *elav>* P[UAS]^218^ flies.
(9.14 MB TIF)

Figure S2 Progressive degeneration in *elav>ena^rev^* individuals does not result in an impaired climbing ability. In a longitudinal assay the geotactic response of flies of the indicated genotypes was examined. The performance in this paradigm decreased as the flies aged for all genotypes; no significant differences were observed between *elav>ena^rev^* and controls. Homozygous *ena^rev^* also displayed a poor response in this paradigm throughout the lifespan.
(0.49 MB TIF)

Table S1 Flies were entrained to 12∶12h LD cycles for 4 days and then released into DD. Free-running behavior was monitored for 10 additional days. Period values were determined using Clocklab employing Chi-Squared periodogram analysis taking into account only rhythmic individuals. The age and number (n) of flies are indicated for each genotype. Percentage of Rhythmic, Weakly Rhythmic and Arrhythmic is also shown. The mean period, the mean power FFT (power FFT is a quantification of the strength of the circadian rhythm) and total activity levels for those flies is shown.
(0.05 MB DOC)

Table S2 Synchronized flies were examined in the automated behavioral paradigm, as explained in the legend to Table S1. The table includes all the experiments described in Figures 3 and 5.
(0.05 MB DOC)
