# Supplementary figures and images for: A Functional Misexpression Screen Uncovers a Role for Enabled in Progressive Neurodegeneration
Source: PLoS One. 2008 Oct 8;3(10):e3332. doi: 10.1371/journal.pone.0003332 (PMC2553195; doi:10.1371/journal.pone.0003332)

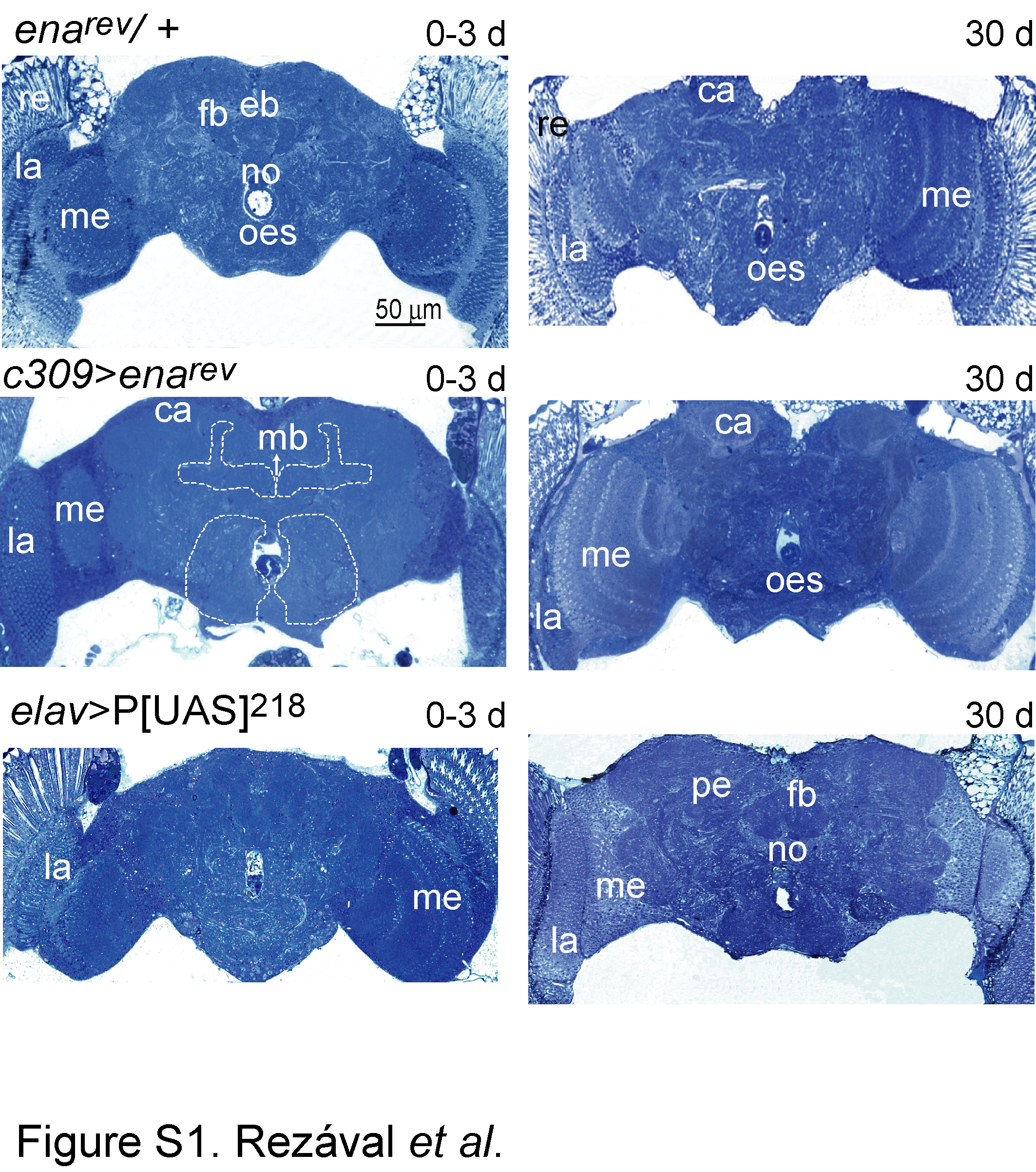

Supplement: Figure S1 — Frontal adult head semi-thin sections (1 µm thick) were stained with methylene blue and examined by light microscopy. Representative sections are included. Young (0–3 day old) and old (30 day old) flies were analyzed for each genotype. Heterozygous ena rev flies show no signs of degeneration throughout adulthood. The C309 driver was employed to reduce ENA levels in the central brain (indicated by the dashed line) but not in the optic lobe. Neither young nor aged C309>enarev flies show any sign of neurodegeneration. The control insertion line P[UAS]218 displaying high levels of rhythmicity at older stages was also evaluated employing the panneural elav driver. No evidence of vacuolization was observed in young or aged elav> P[UAS]218 flies. (9.14 MB TIF) [file pone.0003332.s001.tif]

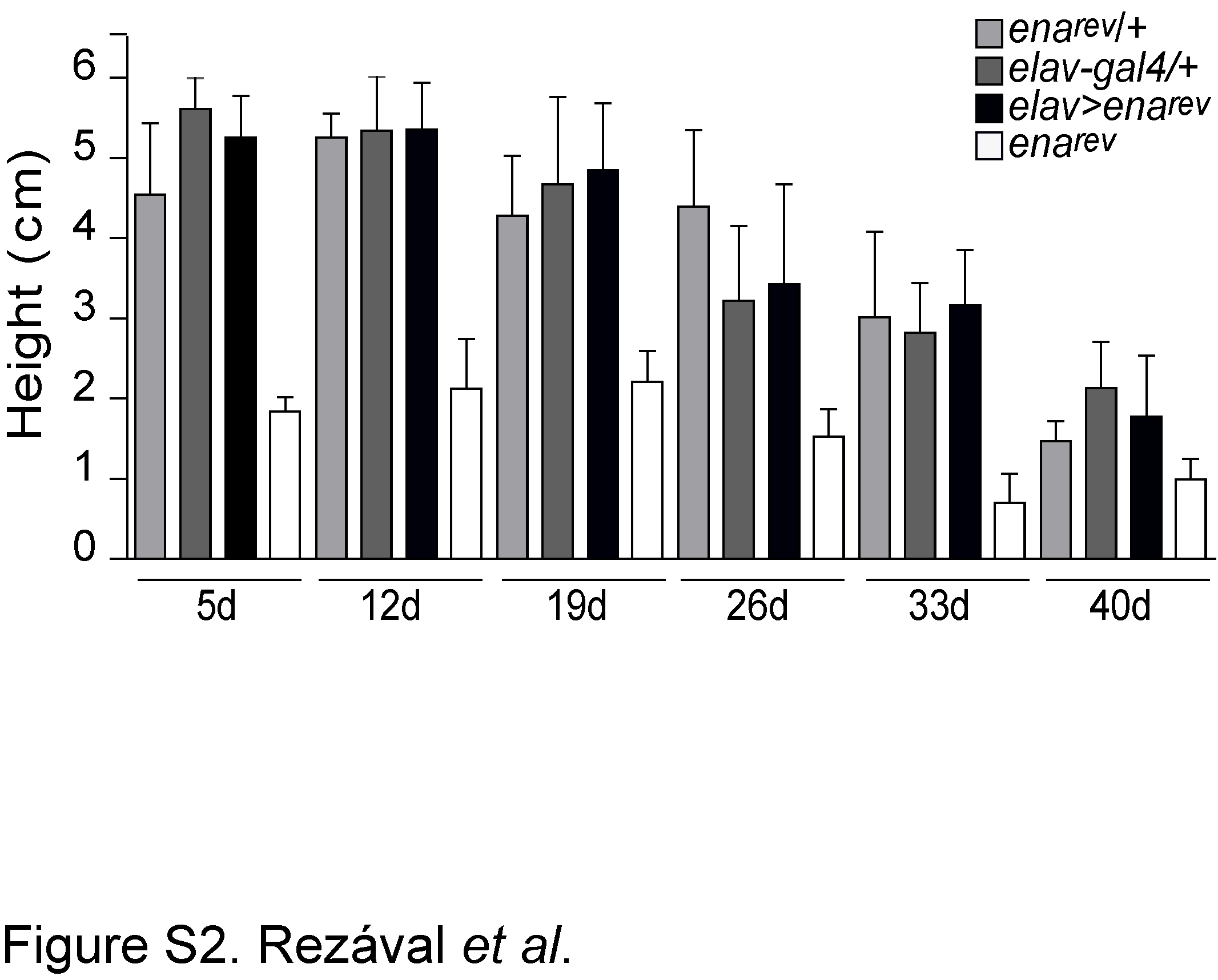

Supplement: Figure S2 — Progressive degeneration in elav>enarev individuals does not result in an impaired climbing ability. In a longitudinal assay the geotactic response of flies of the indicated genotypes was examined. The performance in this paradigm decreased as the flies aged for all genotypes; no significant differences were observed between elav>enarev and controls. Homozygous enarev also displayed a poor response in this paradigm throughout the lifespan. (0.49 MB TIF) [file pone.0003332.s002.tif]
