# Supplementary material for: A Functional Misexpression Screen Uncovers a Role for Enabled in Progressive Neurodegeneration
Source: PLoS One. 2008 Oct 8;3(10):e3332. doi: 10.1371/journal.pone.0003332 (PMC2553195; doi:10.1371/journal.pone.0003332)
Supplement: Table S1 — Flies were entrained to 12∶12h LD cycles for 4 days and then released into DD. Free-running behavior was monitored for 10 additional days. Period values were determined using Clocklab employing Chi-Squared periodogram analysis taking into account only rhythmic individuals. The age and number (n) of flies are indicated for each genotype. Percentage of Rhythmic, Weakly Rhythmic and Arrhythmic is also shown. The mean period, the mean power FFT (power FFT is a quantification of the strength of the circadian rhythm) and total activity levels for those flies is shown. (0.05 MB DOC) [file pone.0003332.s003.doc]

Table S1.

Flies were entrained to 12:12h LD cycles for 4 days and then released into DD. Free-running behavior was monitored for 10 additional days. Period values were determined using Clocklab employing Chi-Squared periodogram analysis taking into account only rhythmic individuals. The age and number (n) of flies are indicated for each genotype. Percentage of Rhythmic, Weakly Rhythmic and Arrhythmic is also shown. The mean period, the mean power FFT (power FFT is a quantification of the strength of the circadian rhythm) and total activity levels for those flies is shown.

|  |  |  |  |  |  |  |  |  |  |  |
| --- | --- | --- | --- | --- | --- | --- | --- | --- | --- | --- |
|  |  |  |  |  |  |  |  |  |  |  |
| Genotype | | Age | n | DD | | | Mean period (h) | FFT | Total Activity |  |
|  |  | (days) |  | % R | % WR | % AR |  |  |  |  |
|  |  |  |  |  |  |  |  |
|  |  |  |  |  |  |  |  |  |  |  |
|  |  | 0-3 | 75 | 82.1 | 16.2 | 1.7 | 23.67 ± 0.05 | 0.15 ± 0.01 | 1566 ± 72.25 |  |
|  |  | 15-18 | 80 | 83.6 | 12.6 | 3.8 | 23.77 ± 0.51 | 0.11 ± 0.01 | 1514 ± 74.89 |  |
| C S | | 30-33 | 78 | 78.6 | 17.8 | 3.6 | 24.10 ± 0.06 | 0.12 ± 0.01 | 1543 ± 181.8 |  |
|  |  | 44-47 | 66 | 66.8 | 24.0 | 9.2 | 24.02 ± 0.09 | 0.13 ± 0.02 | 1710 ± 99.73 |  |
|  |  | 60-63 | 54 | 76.5 | 18.3 | 5.2 | 24.08 ± 0.08 | 0.10 ± 0.01 | 1098 ± 45.40 |  |
|  |  |  |  |  |  |  |  |  |  |  |
|  |  |  |  |  |  |  |  |  |  |  |
|  |  | 0-3 | 66 | 76.0 | 17.5 | 6.5 | 23.89 ± 0.10 | 0.10 ± 0.01 | 1005 ± 60.89 |  |
|  |  | 15-18 | 72 | 73.9 | 24.0 | 2.1 | 24.06 ± 0.10 | 0.10 ± 0.01 | 846.5 ± 46.58 |  |
| *pdf*-*gal4*/+ | | 30-33 | 68 | 75.6 | 24.4 | 0.0 | 24.21 ± 0.10 | 0.10 ± 0.01 | 922.0 ± 52.95 |  |
|  |  | 44-47 | 66 | 64.4 | 26.9 | 8.7 | 24.47 ± 0.10 | 0.08 ± 0.01 | 769.3 ± 70.72 |  |
|  |  | 60-63 | 67 | 68.9 | 26.6 | 4.5 | 24.29 ± 0.09 | 0.08 ± 0.01 | 768.9 ± 50.17 |  |
