# Supplementary material for: A Functional Misexpression Screen Uncovers a Role for Enabled in Progressive Neurodegeneration
Source: PLoS One. 2008 Oct 8;3(10):e3332. doi: 10.1371/journal.pone.0003332 (PMC2553195; doi:10.1371/journal.pone.0003332)
Supplement: Table S2 — Synchronized flies were examined in the automated behavioral paradigm, as explained in the legend to Table S1. The table includes all the experiments described in Figures 3 and 5. (0.05 MB DOC) [file pone.0003332.s004.doc]

Table S2.

Synchronized flies were examined in the automated behavioral paradigm, as explained in the legend to Table S1. The table includes all the experiments described in Figures 3 and 5.

| Genotype | Age (days) | n | %R (DD) |
| --- | --- | --- | --- |
| *pdf-gal4*/+ | 0-3 | 66 | 77.2 |
| *pdf-gal4*/+ | 21 | 54 | 73.2 |
| *pdf>ena^rev^* | 0-3 | 55 | 74.4 |
| *pdf>ena^rev^* | 21 | 89 | 46.2 |
| *ena^rev^/+* | 0-3 | 36 | 88.0 |
| *ena^rev^/+* | 21 | 55 | 78.9 |
| *ena^rev^* | 0-3 | 40 | 60.4 |
| *ena^rev^* | 21 | 86 | 37.4 |
| *ena^rev^/ ena^GC5^* | 0-3 | 36 | 55.1 |
| *ena^rev^/ ena^GC5^* | 21 | 51 | 38.1 |
| *ena*^GC5^/+ | 21 | 18 | 89.6 |
| UAS-*ena*/+ | 24-28 | 30 | 76.4 |
| *pdf-gal4, ena^rev^/+*+ | 24-28 | 71 | 38.2 |
| *pdf-gal4, ena^rev^/*UAS-*ena* | 24-28 | 60 | 66.1 |
| 18105*/+* | 0-3 | 21 | 92.9 |
| 18105*/+* | 21 | 41 | 90.9 |
| 18105*/ena^rev^* | 0-3 | 46 | 100.0 |
| 18105*/ena^rev^* | 21 | 37 | 84.8 |
| *khc^6^/+* | 0-3 | 20 | 90.0 |
| *khc*^6^*/+* | 21 | 52 | 75.5 |
| *khc^6^/ena^rev^* | 0-3 | 48 | 75.8 |
| *khc^6^/ ena^rev^* | 21 | 52 | 57.0 |
